# Increased Resistin Serum Concentrations in Patients with Type 1 Diabetes Mellitus

**DOI:** 10.4274/Jcrpe.1072

**Published:** 2013-09-18

**Authors:** İclal Geyikli, Mehmet Keskin, Yılmaz Kör, Müslüm Akan

**Affiliations:** 1 Gaziantep University, Faculty of Medicine, Department of Biochemistry and Clinical Biochemistry, Gaziantep, Turkey; 2 Gaziantep University, Faculty of Medicine, Pediatric Endocrinology and Metabolisms, Gaziantep, Turkey

**Keywords:** type 1 diabetes mellitus, Adiponectin, leptin, resistin

## Abstract

**Objective:** Adiponectin, leptin, and resistin are adipokines which play a significant role in the regulation of lipid and carbohydrate metabolism in patients with type 2 diabetes, while little is known about their role in type 1 diabetes mellitus (T1DM). The aim of this study was to measure serum adiponectin, leptin, and resistin levels and to investigate their relationships with some parameters in patients with T1DM and healthy controls.

**Methods:** Fifty children and adolescents with T1DM (21 boys and 29 girls) and 33 healthy control subjects (18 boys and 15 girls) participated in the study. All subjects were patients followed in the Pediatric Endocrinology and Metabolism Unit of Gaziantep University Faculty of Medicine. None of the subjects had hypertension, obesity, hyperlipidemia, anemia, or infection. Adiponectin, leptin, and resistin levels were analyzed with ELISA.

**Results:** There were no statistically significant differences related with age, sex, pubertal status, or body mass index distribution between the diabetic and control groups. Resistin levels were significantly higher in the diabetic group compared to controls (5.26±3.15 ng/mL vs. 3.50±1.26 ng/mL; p<0.01).

**Conclusion:** Of the three investigated adipokines, only resistin was associated with T1DM. Resistin may play a role in the process of inflammation and also in the pathophysiology of T1DM.

**Conflict of interest:**None declared.

## INTRODUCTION

Adipose tissue is now known as an active endocrine organ which, in addition to regulating fat mass and nutrient homeostasis, releases a large number of bioactive mediators (adipokines) modulating the homeostasis, blood pressure, lipid and glucose metabolism, inflammation, and inflammation-related diseases such as atherosclerosis (1,2). Adipokines are proteins secreted by the adipose tissue. Leptin and adiponectin, because of their significant role in the regulation of lipid and carbohydrate metabolism, are the two adipokines that have been extensively studied in vitro and in vivo in animals and in human subjects with type 1 (T1DM) and type 2 diabetes mellitus (T2DM). Adiponectin increases insulin sensitivity in the liver and also in the skeletal muscle (3,4,5,6). Leptin decreases appetite, increases energy expenditure, suppresses insulin synthesis and secretion and increases insulin sensitivity (7,8,9,10). Changes in the secretion of or sensitivity to leptin and adiponectin may possibly contribute to the development of T1DM and T2DM.

Resistin was first introduced by Steppan et al (11) as a novel peptide synthesized and secreted from murine adipocytes. Resistin is described as a low molecular-weight (12.5 kDa) adipokine that is a member of a cysteine-rich secretory protein family (12), known as resistin-like molecules (RELMs). The discovery of resistin has led the way to intense research in the area of fat-derived mediators in obesity-induced insulin resistance and T2DM (2). Resistin was initially proposed to be involved in insulin resistance and T2DM. Recently, it was reported to also have a role in inflammation and inflammation-related diseases like atherosclerosis and arthritis (13,14,15).

Besides their other functions, adipokines are involved in inflammatory responses. They exhibit predominantly proinflammatory or anti-inflammatory properties and contribute to insulin resistance. Defects in insulin action and insulin secretion are both present in T2DM. T1DM is characterized by excess inflammation, independent of adiposity and glycemic control. Knowledge about the relationships between adipokines and T1DM is scarce. In this study, serum adiponectin, leptin, and resistin levels were measured in patients with T1DM and in healthy controls, aiming to contribute to the knowledge on their role in the pathogenesis of T1DM. 

## METHODS

Fifty children and adolescents with T1DM (21 boys and 29 girls, mean age 10.43±3.25 years) and 33 healthy control subjects (18 boys and 15 girls, mean age 9.70±4.39 years) participated in the study. All T1DM patients were being followed in the Pediatric Endocrinology and Metabolism Unit of Gaziantep University Faculty of Medicine. Patients with hypertension, obesity, hyperlipidemia, anemia, or infection were not included in the study. Patients with other forms of diabetes (type 2, maturity-onset diabetes of the young, thiamine-responsive megaloblastic anemia) were also excluded. Eligibility criteria for the control group were: 1) prepubertal and pubertal age; 2) absence of acute or chronic inflammatory and autoimmune diseases; 3) lack of diabetes mellitus, primary hyperlipidemia, hypertension, anemia, and obesity; and 4) no current regular medications. The study received approval from the ethics committee of the hospital.

Anthropometric data included height, body weight, and body mass index (BMI). Height was measured in centimeters using a stadiometer. Weight was measured in kilograms using an electronic scale. BMI was calculated using measured weight (kilograms) divided by measured height (meters) squared. Pubertal development was classified according to the Tanner stages. Blood pressure was measured in all subjects, using a standard sphygmomanometer. Blood samples were collected during routine analysis, following an overnight fast. After separation, serum samples were immediately stored at -70°C until analyzed for adipokines. Serum glucose, total cholesterol, high-density lipoprotein (HDL), low-density lipoprotein (LDL), triglyceride, hemoglobin, hematocrit, white blood cell count, free thyroxine (fT4), thyroid-stimulating hormone (TSH), and antithyroid peroxidase (anti-TPO) antibody levels were evaluated by chemical immunoassay method in all patients and controls. Glycosylated hemoglobin (HbA1c) was measured as a marker of glycaemic control in the diabetic patients. HbA1c levels were mathematically standardized to the Diabetes Control and Complications Trial reference range of 4.05-6.05% using multiple of the mean transformation. TPO were defined as positive if higher than 40 U/mL (antibody titre). The diagnosis of Hashimoto thyroiditis was established by demonstrating a high level of TPO antibody. Tissue transglutaminase antibody (tTG) level was analyzed by enzyme-linked immunosorbent assay (ELISA). Celiac disease was suspected if tTG was higher than 15 U.

In this study, adiponectin, leptin and resistin levels were determined with a sandwich ELISA using quantikine ELISA kits. The serum samples have been tested in a blinded fashion. Adiponectin was measured by ELISA (Human Total Adiponectin/Acrp 30 Immunoassay Catalog Number DRP300, Catalog Number SRP300, Catalog Number PDRP300) with intra- and inter-assay coefficients of variation below 5% and 6%, respectively. Leptin was measured by ELISA (Catalog Number DLP00, Catalog Number SLP00, Catalog Number PDLP00) with intra- and inter-assay coefficients of variation below 5% and 4%, respectively. A monoclonal antibody specific for leptin has been pre-coated onto a microplate. Resistin was measured by ELISA (Catalog Number DRSN00, Catalog Number SRSN00, Catalog Number PDRSN00) with intra- and inter-assay coefficients of variation below 4%. A monoclonal antibody specific for resistin has been pre-coated onto a microplate.

**Statistical Analysis**

Analysis was performed using SPSS version 13 software for Windows. Data are reported as means± standard deviation (SD) or medians (25^th^; 75^th^ percentile) for parametric and non-parametric variables, respectively. The differences between the two groups were tested by the t-test for independent samples with normal data distribution or by the Man-Whitney non-parametric test. A p-value of less than 0.05 was regarded as statistically significant. 

## RESULTS

**Figure 1:**
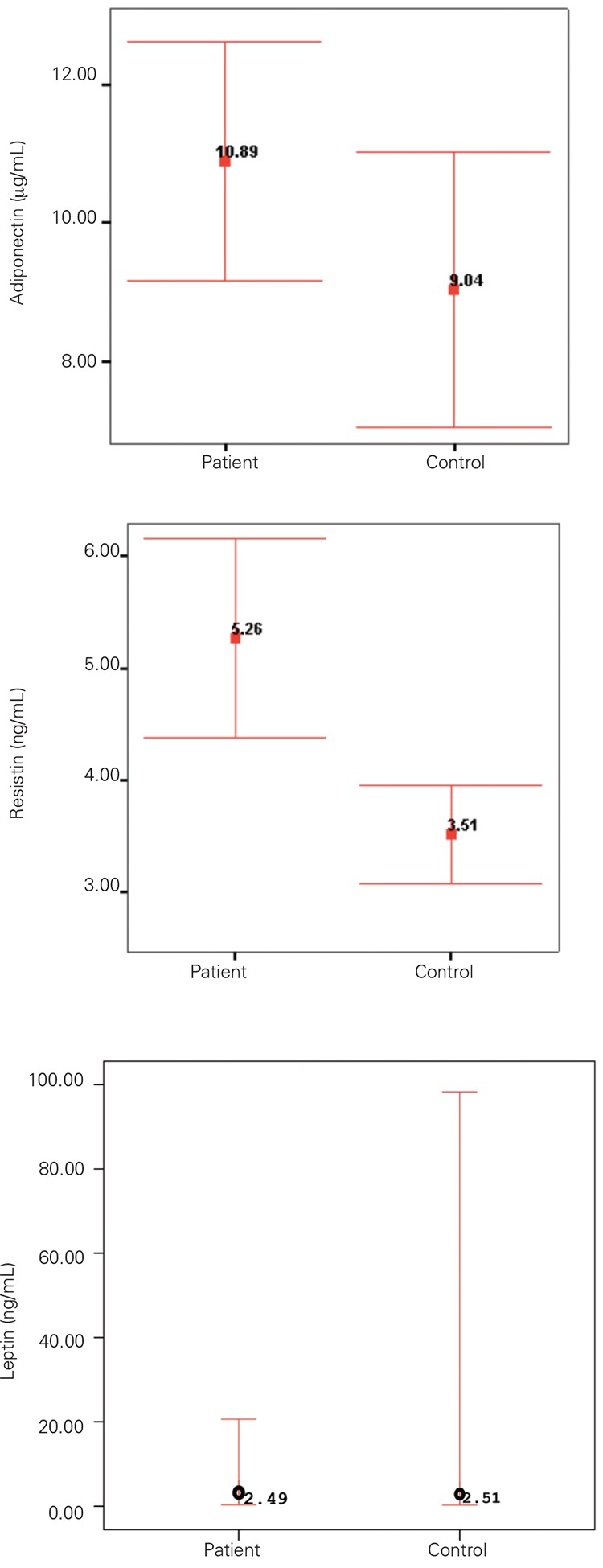
Mean (±SD) values of adiponectin, resistin and median (25th; 75th percentile) value of leptin levels in children with type 1 diabetes mellitus and age-and sex-matched controls

The male/female ratio of the diabetic group was 21/29. Mean diabetic duration was 2.96±2.63 years. Demographic and clinical characteristic of the diabetic patients are shown in Table 1. In the control group, male/female ratio was 18/15 and mean age was 9.70±4.39 years. There were no statistically significant differences in age, sex, or pubertal status between the patient and control groups. BMI distribution between the diabetic and control groups was similar (p>0.05). Resistin levels were significantly higher in the diabetic group compared to controls (5.26±3.15 ng/mL vs. 3.50±1.26 ng/mL; p<0.01). There were no significant differences in mean adiponectin (10.88±5.98µg/mL vs. 9.04±5.69 µg/mL) and median (interquartile) leptin (2.48 ng/mL vs. 2.51 ng/mL) levels between the two groups. Mean (±SD) values for adiponectin, resistin, and median (25^th^; 75^th^ percentile) values for leptin levels in children with T1DM and age-and sex-matched controls are shown in Figure 1. Analysis investigating the correlation of adipokine levels with HbA1c, diabetes duration, BMI, and age showed a positive correlation between HbA1c and resistin levels (r=0.321, p=0.03). Adipokines were not interrelated. 

## DISCUSSION

T1DM is a T cell-mediated autoimmune disease characterized by excess inflammation, independent of adiposity and glycemic control. The incidence of T1DM is increasing at 3-5% per year worldwide (16,17,18), and this increase cannot be accounted for by known genetic factors. Several acute-phase inflammatory markers have been reported to be increased in T1DM. Little information is available on the different patterns of T1DM progression following diagnosis, particularly in the pediatric population. Systemic cytokines, which are known to play a role in the autoimmune process leading to destruction of beta cells (19,20), could be potential biomarkers for different patterns of disease progression. In this study, we investigated the relationships of adiponectin, leptin, and resistin levels with other parameters in pediatric diabetic patients and healthy controls.

The adipose tissue is increasingly recognized to be not only a storage organ for lipids but rather a metabolically highly active endocrine organ. Studies have revealed that adipocytes synthesize and secrete a number of biologically active molecules (21). The adipose tissue regulates skeletal muscle insulin sensitivity through a number of circulating adipocyte- derived hormones, the so called adipocytokines, including tumor necrosis factor-α (TNF-α), leptin, interleukin-6 (IL-6), plasminogen activator inhibitor-1 (PAI-1), adiponectin, and resistin, which act locally and distally through their autocrine, paracrine, and endocrine effects (1,22). Among these, adiponectin is the most abundant and is shown to have insulin-sensitizing, antiatherogenic, and anti-inflammatory properties (4,5). In addition to its insulin-sensitizing effects, adiponectin may alter glucose metabolism through stimulation of pancreatic insulin secretion in vivo. In humans, plasma adiponectin levels were correlated negatively with adiposity (3,4,5), insulin resistance (5,6,23), T2DM (5), and metabolic syndrome (24).

Leptin is a metabolic protein produced by the adipose tissue, and both leptin and leptin receptor levels have been reported to be increased in individuals with T1DM (25). Leptin serves as a major ‘adipostat’ by repressing food intake and promoting energy expenditure. Independent of these effects, leptin improves peripheral (hepatic and skeletal muscle) insulin sensitivity and modulates pancreatic β-cell function. Leptin regulates food intake via brain signaling of satiety and energy store levels and is paradoxically increased in obesity, with obese individuals appearing to be resistant to the effects of leptin (7,8,9,10). Concentrations of leptin are reported to be higher around puberty and during adolescence (26). In addition to its role in regulating metabolism, leptin has been recognized more recently as a proinflammatory agent (27), and leptin levels have been shown to regulate inflammatory processes and immune cells (28).

In this study, we found that there were no significant differences in mean adiponectin and median leptin levels between the diabetic and control groups. No patients or control subjects had hypertension, obesity, or hyperlipidemia.

Resistin is a secretory protein produced by adipocytes (11). Resistin was first identified in a screen for proteins that were down-regulated by the insulin-sensitizing antidiabetic drug rosiglitazone, and it was shown to cause insulin resistance (11). Resistin plays a role in glucose homeostasis both in mice and humans (29). It is also thought to represent a novel cytokine (30) and to be involved in inflammation (13,14,15). Resistin is measurable in mouse and human serum (11), but data regarding human serum resistin levels and its metabolic regulation are very limited. In this study, we therefore investigated whether human resistin levels are affected by T1DM associated with autoimmunity/inflammation.

Resistin has been reported to cause insulin resistance in animal models, which fueled the hypothesis that this hormone may play a role in the pathogenesis of obesity, mediated insulin resistance, and diabetes. However, the structure and biology of resistin differ substantially between species, and many aspects, specifically its association with obesity and its effects on insulin sensitivity in humans, remain controversial. In contrast to mice, human resistin is expressed at lower levels in adipocytes but at higher levels in circulating blood monocytes (31). The accumulation of macrophages in adipose tissue, the common origin of macrophages and adipocytes, the prevalent presence of peripheral mononuclear cells, and apoptotic beta cells by themselves seem to be the sources of inflammation present in T2DM, since they generate the mediators of the inflammatory processes, namely cytokines. Resistin induced the expression of TNF-α and IL-6 in white adipose tissue and in peripheral-blood mononuclear cells (12,32). Plasma resistin levels were reported to be associated with many inflammatory markers including C-reactive protein (33), soluble TNF-α receptor-2,IL-6, and lipoprotein-associated phospholipase A2 (13) in several pathophysiological conditions. Resistin was shown to be associated with disease activity in patients with inflammatory bowel disease (14), to correlate with severity of disease in severe sepsis and septic shock (34), and to be associated with coronary artery disease (13). Furthermore, resistin may be involved in the pathogenesis of rheumatoid arthritis (12). Considering the crosstalk between inflammatory pathways and the insulin signaling cascade, resistin may represent a link between inflammation and metabolic signal.

This study showed that resistin levels were significantly higher in the diabetic group as compared to controls. An increased level of resistin was also found in patients with high levels of HbA1c. None of our T1DM patients suffered from any other metabolic problem. Hyperglycemia and a worsening of glycemic control in T1DM have been associated with increased inflammation. Resistin may have a role in inflammatory pathways. Resistin is produced and secreted mainly by mononuclear cells in peripheral blood, and hyperglycemia-induced monocyte/macrophage activation may lead to increased secretion of resistin. It is also possible that resistin interferes with the ability of insulin in disposing of glucose from the blood stream. In short, the available literature and also our findings indicate that resistin may possibly be involved in the pathophysiology of T1DM, a conclusion that needs to be supported by new studies.

## Figures and Tables

**Tablo 1:**
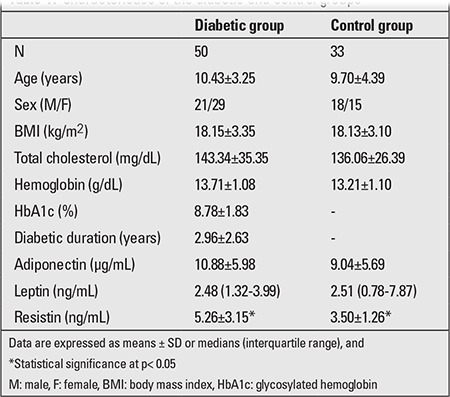
Characteristics of the diabetic and control groups
